# Supplementary material for: Rewiring capsule production by CRISPRi-based genetic oscillators demonstrates a functional role of phenotypic variation in pneumococcal-host interactions
Source: bioRxiv. 2023 Jun 3:2023.06.03.543575. Preprint. [Version 1] doi: 10.1101/2023.06.03.543575 (PMC10312626; doi:10.1101/2023.06.03.543575)
Supplement: Supplement 2 [file media-2.pdf]

Table S1: Strains and plasmids used in this study

Strain	Genotype	Reference
VL1	D39V, Serotype 2 strain	[1]
VL333	D39V, Δ*prs1*::P_{F6}-*lacI*-*tetR* (*gen*)	[2]
VL783	D39V, *comC::ery*	[3]
VL995	D39V, *Δprs1*::P_{F6}-*lacI* (*gen*), *bgaA*::P_{lac}-*dCas9sp* (*tet*), *cil*::P_3-*luc* (*kan*), *cep*::P_3-*sgRNAluc1* (*spc*)	[4]
VL996	D39V, *Δprs1*::P_{F6}-*lacI* (*gen*), *bgaA*::P_{lac}-*dCas9sp* (*tet*), *cil*::P_3-*luc* (*kan*), *cep*::pPEPX (*spc*)	[4]
VL997	D39V, Δ*prs1*::P_{F6}-*lacI* (*gen*), *bgaA*::P_{lac}-*dCas9sp* (*tet*), *cil*::P_3-*luc* (*kan*)	[4]
VL1998	DCI23, Δ*prs1*::P_{F6}-*lacI* (*gen*), *bgaA*::P_{lac}-*dCas9sp* (*tet*)	[4]
VL2969	D39V, Δ*ply*::*camR*, *cil*::*kanR*	Veening lab collection
VL3251	D39V, *Δprs1*::P_{F6}-*lacI* (*gen*), *bgaA*::P_{lac}-*dCas9sp* (*tet*), *cil*::P_3-*luc* (*kan*), *cep*::P_3-BS1-sgRNA*luc* (*spc*)	This study
VL3252	D39V, *Δprs1*::P_{F6}-*lacI* (*gen*), *bgaA*::P_{lac}-*dCas9sp* (*tet*), *cil*::P_3-*luc* (*kan*), *cep*::P_3- BS2-sgRNA*luc* (*spc*)	This study
VL3253	D39V, *Δprs1*::P_{F6}-*lacI* (*gen*), *bgaA*::P_{lac}-*dCas9sp*	This study

	(*tet*), *cil*::P_3-*luc* (*kan*), *cep*::P_3- BS3-sgRNA*luc* (*spc*)	
VL3254	D39V, *Δprs1*::P_{F6}-*lacI* (*gen*), *bgaA*::P_{lac}-*dCas9sp* (*tet*), *cil*::P_3-*luc* (*kan*), *cep*::P_3- BS4-sgRNA*luc* (*spc*)	This study
VL3255	D39V, *Δprs1*::P_{F6}-*lacI* (*gen*), *bgaA*::P_{lac}-*dCas9sp* (*tet*), *cil*::P_3-*luc* (*kan*), *cep*::P_3- BS5-sgRNA*luc* (*spc*)	This study
VL3256	D39V, *Δprs1*::P_{F6}-*lacI* (*gen*), *bgaA*::P_{lac}-*dCas9sp* (*tet*), *cil*::P_3-*luc* (*kan*), *cep*::P_3- BS6-sgRNA*luc* (*spc*)	This study
VL3308	D39V, *Δprs1*::P_{F6}-*lacI-tetR* (*gen*), *bgaA*::P_{lac}-*dCas9sp* (*tet*)	This study
VL3309	D39V, Δ*prs1*::P_{F6}-*lacI* (*gen*), *zip*::P_{lac}-*mNeonGreen*-opt-TEV-*mScarletI-opt* (*spc*)	Veening lab collection
VL3312	D39V, Δ*prs1*::P_{F6}-*lacI* (*gen*), *zip*::P_{lac}-*mNeonGreen-opt*-TEV-*mTurquoise2-opt* (*spc*)	Veening lab collection
VL3436	D39V, *Δprs1*::P_{F6}-*lacI-tetR* (*gen*), *bgaA*::P_{lac}-*dCas9sp* (*tet*), *cep*::P_3-BS3-sgRNA2 (*spc*)	This study

VL3437	D39V, *Δprs1*::P_{F6}-*lacI-tetR* (*gen*), *bgaA*::P_{lac}-*dCas9sp* (*tet*), *cep*::P_3-BS6-sgRNA3 (*spc*)	This study
VL3438	D39V, *Δprs1*::P_{F6}-*lacI-tetR* (*gen*), *bgaA*::P_{lac}-*dCas9sp* (*tet*), *cep*::P_3-BS2-sgRNA6 (*spc*)	This study
VL3439	D39V, *Δprs1*::P_{F6}-*lacI-tetR* (*gen*), *bgaA*::P_{lac}-*dCas9sp* (tet),<br>*cep*::P_3-BS3-sgRNA2 (*spc*), *zip*::P_3-BS2-*mNeonGreen-opt* (*ery*)	This study
VL3440	D39V, *Δprs1*::P_{F6}-*lacI-tetR* (*gen*), *bgaA*::P_{lac}-*dCas9sp* (*tet*),<br>*cep*::P_3-BS3-sgRNA2 (*spc*), *zip*::P_3-BS2-*mNeonGreen-opt* (*ery*)	This study
VL3441	D39V, *Δprs1*::P_{F6}-*lacI-tetR* (*gen*), *bgaA*::P_{lac}-*dCas9sp* (*tet*),<br>cep::P_3-BS2-sgRNA6 (*spc*), zip::P_3-BS6-*mScarletI-opt* (*ery*)	This study
VL3660	D39V, *Δcps*	Veening lab collection
VL3703	D39V, P_{cps}::*tetM-tetR*-P_{tet}-*cps* (*tet*)	[2]
VL3746<br>(Strain 1)	D39V, *Δprs1*::P_{F6}-*lacI-tetR* (*gen*), *bgaA*::P_{lac}-*dCas9sp* (*tet*), *zip*::P_3-BS6-sgRNA3-P_3-BSA2-*mNeonGreen-opt* (*ery*)	This study

VL3747<br>(Strain 2)	D39V, *Δprs1*::P_{F6}-*lacI*-*tetR* (*gen*), *bgaA*::P_{lac}-*dCas9sp* (tet), *cep*::P_3-BS6-*mScarletI-opt*-P_3-BS3-sgRNA2 (*spc*)	This study
VL3748<br>(Strain 3)	D39V, *Δprs1*::P_{F6}-*lacI*-*tetR* (*gen*), *bgaA*::P_{lac}-*dCas9sp* (tet), *cil*::P_3-BS3-*mTurquoise2-opt*-P_{tet}-BS2-sgRNA6 (*kan*)	This study
VL3749<br>(Strain 4)	D39V, *Δprs1*::P_{F6}-*lacI*-*tetR* (*gen*), *bgaA*::P_{lac}-*dCas9sp* (*tet*), *cil*::P_3-BS3-*mTurquoise2-opt*-P_3-BS2-sgRNA6 (*kan*)	This study
VL3750<br>(Int Strain)	D39V, *Δprs1*::P_{F6}-*lacI*-*tetR* (*gen*), *bgaA*::P_{lac}-*dCas9sp* (*tet*), *zip*::P_3-BS6-sgRNA3-P_3-BS2-*mNeonGreen-opt* (*ery*), *cep*::P_3- BS6-*mScarletI-opt*-P_3-BS3-sgRNA2 (*spc*)	This study
VL3752<br>(Strain 5)	D39V, *Δprs1*::P_{F6}-*lacI*-*tetR* (*gen*), *bgaA*::P_{lac}-*dCas9sp* (*tet*), *zip*::P_3-BS6-sgRNA3-P_3-BS2-*mNeonGreen-opt* (*ery*), *cep*::P_3- BS6-*mScarletI-opt*-P_3-BS3-sgRNA2 (*spc*), *cil*::P_3-BS3-*mTurquoise2-opt*-P_{tet}-BS2-sgRNA6 (*kan*)	This study
VL3753<br>(Strain 6)	D39V, *Δprs1*::P_{F6}-*lacI*-*tetR* (*gen*), *bgaA*::P_{lac}-*dCas9sp* (*tet*), *zip*::P_3-BS6-sgRNA3-P_3-BS2-*mNeonGreen-opt* (*ery*),	This study

	cep::P_3- BS6-*mScarletI-opt*-P_3-BS3-sgRNA2 (*spc*), *cil*::P_3-BS3-*mTurquoise2-opt*-P_3-BS2-sgRNA6 (*kan*)	
VL3755<br>(Strain5 +AC)	D39V, *Δprs1*:: P_{F6}-*lacI-tetR* (*gen*), *bgaA*::Plac-*dCas9*sp (*tet*), *zip*::P_3-BS6-sgRNA3-P_3-BS2-*mNeonGreen-opt* (*ery*), *cep*::P_3-BS6-*mScarletI-opt*-P_3-BS3-sgRNA2 (*spc*), *cil*::P_3-BS3-*mTurquoise2-opt*-P_{tet}-BS2-sgRNA6 (*kan*), *lytA*::*cat*, *comC*::P_{comC}-*tmp*	This study
VL3757<br>(Strain6 +AC)	D39V, *Δprs1*::P_{F6}-*lacI-tetR* (*gen*), *bgaA*::P_{lac}-*dCas9sp* (*tet*), *zip*::P_3-BS6-sgRNA3-P_3-BS2-*mNeonGreen-opt* (*ery*), *cep*::P_3- BS6-*mScarletI-opt*-P_3-BS3-sgRNA2 (*spc*), *cil*::P_3-BS3-*mTurquoise2-opt*-P_3-BS2-sgRNA6 (*kan*), *lytA::cat*, *comC*::P_{comC}-*tmp*	This study
VL3869<br>(VL3755+del-cps)	D39V, *Δprs1*:: P_{F6}-*lacI-tetR* (*gen*), *bgaA*::Plac-*dCas9*sp (*tet*), *zip*::P_3-BS6-sgRNA3-P_3-BS2-*mNeonGreen-opt* (*ery*), *cep*::P_3-BS6-*mScarletI-opt*-P_3-BS3-sgRNA2 (*spc*), *cil*::P_3-BS3-*mTurquoise2-opt*-P_{tet}-BS2-sgRNA6 (*kan*), *lytA::cat*, *comC*::P_{comC}-*tmp, Δcps*	This study

VL3870	D39V, *bgaA::*P$_{lac}$-*dcas9sp* (*gen*)	This study
VL3872	D39V, P$_{cps}$::*tetM*-*tetR*-P$_3$-BS6-*mScarletI-opt-cps* (*tet*)	This study
VL3873	D39V, P$_{cps}$::*tetM*-*tetR*-P$_3$-BssgRNA6-*mScarletI-opt-cps* (*tet*), *cep*::P$_3$-BS3-sgRNA2 (*spc*)	This study
VL3875	D39V, P$_{cps}$::*tetM*-*tetR*-P$_3$-BS6-*mScarletI-opt-cps* (*tet*), *cep*::P$_3$-BS3-sgRNA2 (*spc*), *zip*::P$_3$-BS6-sgRNA3-P$_3$-BS2-*mNeonGreen-opt* (*ery*)	This study
VL3876	D39V, P$_{cps}$::*tetM*-*tetR*-P$_3$-BS6-*mScarletI-opt-cps* (*tet*), *cep*::P$_3$-BS3-sgRNA2 (*spc*), *zip*::P$_3$-BS6-sgRNA3-P$_3$-BS2-*mNeonGreen-opt* (*ery*), *cil*::P$_3$-BS3-*mTurquoise2-opt*-P$_{tet}$-BS2-sgRNA6 (*kan*)	This study
VL3877	D39V, P$_{cps}$::*tetM*-*tetR*-P$_3$-BS6-*mScarletI-opt-cps* (*tet*), *cep*::P$_3$-BS3-sgRNA2 (*spc*), *zip*::P$_3$-BS6-sgRNA3-P$_3$-BS2-*mNeonGreen-opt* (*ery*), *cil*::P$_3$-BS3-*mTurquoise2-opt*-P$_3$-BS2-sgRNA6 (*kan*)	This study
VL3878	D39V, P$_{cps}$::*tetM*-*tetR*-P$_3$-BS6-*mScarletI-opt-cps* (*tet*), *cep*::P$_3$-BS3-sgRNA2 (*spc*), *zip*::P$_3$-	This study

	BS6-sgRNA3-P_3-BS2-*mNeonGreen-opt* (*ery*), *cil*::P_3-BS3-*mTurquoise2-opt*-P_{tet}-BS2-sgRNA6 (*kan*), *bgaA*::P_{lac}-*dcas9sp* (*gen*)<br><br>Inducible CAPSUlator 1.0	
VL3879	D39V, P_{cps}::*tetM-tetR*-P_3-BS6-*mScarletI-opt-cps* (*tet*), *cep*::P_3-BS3-sgRNA2 (*spc*), *zip*::P_3-BS6-sgRNA3-P_3-BS2-*mNeonGreen-opt* (*ery*), *cil*::P_3-BS3-*mTurquoise2-opt*-P_3-BS2-sgRNA6 (*kan*), *bgaA*::P_{lac}-*dcas9sp* (*gen*)<br><br>Constitutive CAPSUlator 1.0	This study
VL3882	D39V, *Δprs1::P_{F6}-lacI (gen), zip::tmp-P_{lac}-MCS (tmp)*	Veening lab collection
**VL4313**	D39V, *zip*:: P_3- BS6-sgRNA3- P_3-BS2-*mNeonGreen-opt* (*ery*)	This study
VL4315	D39V, *zip*:: P_3- BS6-sgRNA3- P_3-BS2-*mNeonGreen-opt* (*ery*), P_{cps}::*tetM-tetR*-P_3-BS6-*mScarletI-opt-cps* (*tet*), *cep*:: P_3-BS3-sgRNA2 (*spc*) , *bgaA*::P_{lac}-dCas9-P_3-BS3-*mTurquoise2-opt*-P_3-BS2-sgRNA6 (*gen*)<br><br>CAPSUlator 2.0	This study
VL4316	D39V, *zip*:: P_3- BS6-sgRNA3- P_3-BS2-*mNeonGreen-opt* (*ery*),	This study

	P_{cps}::*tetM-tetR*-P_3-BS6-*mScarletI-opt*-Δ*cps* (*tet*) (NO *cps*)	
VL4317	D39V, *zip*:: P_3- BS6-sgRNA3- P_3-BS2-*mNeonGreen-opt* (*ery*), P_{cps}::*tetM-tetR*-P_3-BS6-*mScarletI-opt*-Δ*cps* (*tet*) (NO *cps*), *cep*:: P_3-BS3-sgRNA2 (*spc*)	This study
VL4318	D39V, *zip*:: P_3- BS6-sgRNA3- P_3-BS2-*mNeonGreen-opt* (*ery*), P_{cps}::*tetM-tetR*-P_3-BS6-*mScarletI-opt-cps* (*tet*)	This study
VL4319	D39V, *zip*:: P_3- BS6-sgRNA3- P_3-BS2-*mNeonGreen-opt* (*ery*), P_{cps}::*tetM-tetR*-P_3-BS6-*mScarletI-opt-cps* (*tet*), *cep*:: P_3-BS3 (*spc*) (NO sgRNA2)	This study
VL4320	D39V, *zip*:: P_3- BS6-sgRNA3- P_3-BS2-*mNeonGreen-opt* (*ery*), *cep*:: P_3-BS3-sgRNA2 (*spc*)	This study
VL4321	D39V, *zip*:: P_3- BS6-sgRNA3- P_3-BS2-*mNeonGreen-opt* (*ery*), P_{cps}::*tetM-tetR*-P_3-BS6-*mScarletI-opt*-Δ*cps* (*tet*) (NO *cps*), *cep*:: P_3-BS3-sgRNA2 (*spc*), *bgaA*::P_{lac}-dCas9-P_3-BS3-*mTurquoise2-opt*-P_3-BS2-sgRNA6 (*gen*)<br>CAPSU2-Ctl-Delta-cps	This study

VL4322	D39V, *zip*:: P_3- BS6-sgRNA3- P_3-BS2-*mNeonGreen-opt* (*ery*), P_{cps}::*tetM-tetR*-P_3-BS6-*mScarletI-opt-cps* (*tet*), *cep*:: P_3-BS3 (*spc*) (NO sgRNA2), *bgaA*::P_{lac}-dCas9-P_3-BS3-*mTurquoise2-opt*-P_3-BS2-sgRNA6 (*gen*)<br>CAPSU2-Ctl-Always-OFF	This study
VL4323	D39V, *zip*:: P_3- BS6-sgRNA3- P_3-BS2-*mNeonGreen-opt* (*ery*), P_{cps}::*tetM-tetR*-P_3-BS1-*mScarletI-opt-cps* (*tet*) (NO BS6 but BS1), *cep*:: P_3-BS3-sgRNA2 (*spc*)	This study
VL4324	D39V, *zip*:: P_3- BS6-sgRNA3-P_3-BS2-*mNeonGreen-opt* (*ery*), P_{cps}::*tetM-tetR*-P_3-BS1-*mScarletI-opt-cps* (*tet*) (NO BS6 but BS1), *cep*::P_3-BS3-sgRNA2 (*spc*), *bgaA*::P_{lac}-dCas9-P_3-BS3-*mTurquoise2-opt*-P_3-BS2-sgRNA6 (*gen*)<br>CAPSU2-Ctl-Always-ON	This study
**Plasmid**	**Genotype**	**Reference**
pVL1305	pPEPX-P_3-sgRNA*luc, spc*	Veening lab collection
pASR102	pPEPZ-P_{lac}, *gen*	Keller et al
pASR103	pPEPZ-P_{lac}, *ery*, *bla*	Keller et al

pASR110	pPEPZ-P_{lac}-*mNeonGreen-opt*, *spc*	Veening lab collection

References:

1. Slager, J., Aprianto, R. & Veening, J.-W. Deep genome annotation of the opportunistic human pathogen *Streptococcus pneumoniae* D39. *Nucleic Acids Res.* **46**, 9971–9989 (2018).
2. Sorg, R. A., Gallay, C., Van Maele, L., Sirard, J.-C. & Veening, J.-W. Synthetic gene-regulatory networks in the opportunistic human pathogen *Streptococcus pneumoniae*. *Proc Natl Acad Sci U S A* **117**, 27608–27619 (2020).
3. Moreno-Gámez, S. *et al.* Quorum sensing integrates environmental cues, cell density and cell history to control bacterial competence. *Nat Commun* **8**, 854 (2017).
4. Liu, X. *et al.* High-throughput CRISPRi phenotyping identifies new essential genes in *Streptococcus pneumoniae*. *Mol. Syst. Biol.* **13**, 931 (2017).
